# Optimizing cancer therapy: a review of the multifaceted effects of metronomic chemotherapy

**DOI:** 10.3389/fcell.2024.1369597

**Published:** 2024-05-15

**Authors:** Oyku Yagmur Basar, Sawsan Mohammed, M. Walid Qoronfleh, Ahmet Acar

**Affiliations:** ^1^ Department of Biological Sciences, Middle East Technical University, Ankara, Türkiye; ^2^ Qatar University, QU Health, College of Medicine, Doha, Qatar; ^3^ Q3 Research Institute (QRI), Research and Policy Division, Ypsilanti, MI, United States

**Keywords:** cancer, metronomic chemotherapy, therapeutic resistance, combination therapy, photodynamic therapy

## Abstract

Metronomic chemotherapy (MCT), characterized by the continuous administration of chemotherapeutics at a lower dose without prolonged drug-free periods, has garnered significant attention over the last 2 decades. Extensive evidence from both pre-clinical and clinical settings indicates that MCT induces distinct biological effects than the standard Maximum Tolerated Dose (MTD) chemotherapy. The low toxicity profile, reduced likelihood of inducing acquired therapeutic resistance, and low cost of MCT render it an attractive chemotherapeutic regimen option. One of the most prominent aspects of MCT is its anti-angiogenesis effects. It has been shown to stimulate the expression of anti-angiogenic molecules, thereby inhibiting angiogenesis. In addition, MCT has been shown to decrease the regulatory T-cell population and promote anti-tumor immune response through inducing dendritic cell maturation and increasing the number of cytotoxic T-cells. Combination therapies utilizing MCT along with oncolytic virotherapy, radiotherapy or other chemotherapeutic regimens have been studied extensively. This review provides an overview of the current status of MCT research and the established mechanisms of action of MCT treatment and also offers insights into potential avenues of development for MCT in the future.

## 1 Metronomic chemotherapy: less is more

The principle of chemotherapeutic drugs is to inhibit cell proliferation or induce cell death through many different mechanisms (Shewach and Kuchta, 2009). The traditional regimen for chemotherapeutic administration has predominantly favored the Maximum Tolerated Dose (MTD) therapy approach for a long time (McConnell, 1989). MTD therapy utilizes the maximum dose that can be tolerated by the patient without inducing lethal consequences to the patient (Takimoto, 2009). Though it is aimed at maximizing tumor depletion in the patient, the cytotoxic burden of this treatment option in normal tissues brings out the need for prolonged rest periods between administrations (Xu et al., 2021). Side effects such as extensive damage to healthy tissues and neurotoxic properties are of great concern and greatly impair the quality of life of the patients (Ho et al., 2018). Although this administration route results in a serious decrease in the tumor volume initially, it is often accompanied by the development of therapeutic resistance and subsequent relapse in later stages (Kareva et al., 2015).

The history of MTD being the hallmark of chemotherapeutic treatment dates back to the 1970s when it was proven to be successful in treating pediatric acute lymphoblastic leukemia (Skipper et al., 1970). This was a rare case since the leukemic tumor clone could be fully eliminated. However, this is not the case in many other cancer subtypes, even in leukemias other than acute lymphoblastic leukemia. Many other cancer types require more than just the elimination of the tumor cells using very high MTD doses since there are also many other players contributing to tumorigenesis such as the tumor microenvironment (Wu and Dai, 2017), immune cells (Gun et al., 2019), different cancer subclones (Zhang et al., 2022), and epigenetic alterations (Ilango et al., 2020). Cancer is not a simple disease that can be explained by the mutations of the cancer cells by itself but rather it is a complex interplay revolving around many different aspects (Hanahan and Weinberg, 2011). The philosophy that “the more is better’’ in the case of cancer treatment is outdated, as it has been proven that this approach is not only insufficient but also could be even more harmful, in most cases (Scharovsky et al., 2009). Therefore, the need for the development of different chemotherapeutic administration regimens is of utmost importance.

A novel alternative concept of drug administration regimen named metronomic chemotherapy (MCT) has emerged in the last 2 decades. MCT refers to the continuous administration of low-dose chemotherapeutic agents within close intervals, without long rest periods (Cazzaniga et al., 2016). In the year 2000, Browder et al. published a study exploring the anti-angiogenic effects of varying doses of different cytotoxic drugs, primarily cyclophosphamide on a mouse model. They found that when the drug was given at a lower dose within 6-day intervals, it was more effective than the traditional MTD regimen in terms of tumor shrinkage and anti-angiogenesis properties (Browder et al., 2000). Klement et al. also published a study in the same year on the effects of the drugs Vinblastine and mAb DC-101 which were administered regularly in a mouse model at lower doses than MTD. They found that when these two drugs were given together in lower doses, namely, the MCT regimen, the tumors significantly regressed, anti-angiogenic effects were detected, no relapse period was observed afterward and the toxicity of the treatment was far less than that of MTD regimen (Klement et al., 2000). Later that year, Hanahan et al., used the term “metronomic chemotherapy’’, for the first time in a commentary based on the two studies mentioned above (Hanahan et al., 2000). Since then, it has been backed up by many other preclinical studies as well as clinical trials that MCT has different biological effects than MTD, and the many aspects of MCT have been delineated (Kamat et al., 2007; Schito et al., 2020; Mundo et al., 2022).

**FIGURE 1:**
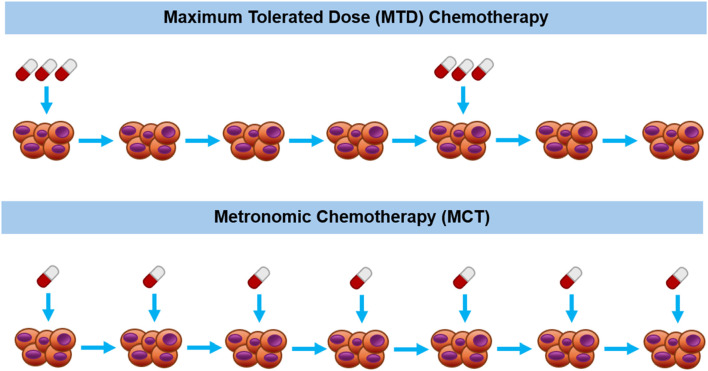
Diagrammatic description of maximum tolerated dose therapy (upper part) vs metronomic therapy (bottom part) for chemotherapeutic drug administration schedules.

The main differences between conventional MTD chemotherapy and MCT lie in the temporal administration regimen and the given dosage of the chemotherapeutic drug. These differences are diagrammatically depicted in Figure 1. In conventional chemotherapy schedules, the standard of care MTD therapy involves administrating the maximum dose that can be tolerated by the patient without the induction of lethal outcomes (Scharovsky et al., 2020). Conversely, in MCT studies, dosages have consistently been reported at levels notably lower than the maximum tolerated dose (Mpekris et al., 2021; Romiti et al., 2017; Wysocki et al., 2022). Another significant difference between conventional MTD chemotherapy and MCT is their temporal administration routes. In conventional MTD chemotherapy, prolonged drug-free periods are often necessitated since the administration of high doses of the chemotherapeutics frequently leads to damage in normal tissues and requires recovery time (Scharovsky et al., 2020). In contrast, MCT employs lower doses, resulting in reduced damage to normal tissues, thereby the drugs can be administrated continuously or with minimal drug-free periods (Shi et al., 2019).

There are a number of therapeutic options available for the treatment of different cancer types. Unfortunately, the opportunity to access them is not the same for people all over the world. According to the 2020 World Health Organization (WHO) report, cancer was responsible for approximately 10 million deaths across the world (World Health Organization, 2020). Among these 10 million cancer-associated deaths, around 70% were from low and middle-income countries (LMICs). Moreover, the incidence rates of cancers were lower in these countries despite the enormous death rate, indicating the necessity for improved monitoring of cancers. Cancer care is associated with an overwhelming demand for resources, infrastructure, and challenges for the national health systems (Fousseyni et al., 2011; Chalkidou et al., 2014) Patients from LMICs lack access to screening, early diagnosis, treatment facilities, pain-relief options, cancer-preventative vaccines, and many more (André et al., 2013). Furthermore, the escalating cost of novel therapeutics renders the clinical trials and biomedical research on the subject unsustainable and slows down scientific progress (Schrag, 2004). Under these conditions, considering the cost of treatment options to provide treatments as accessible as possible hold critical importance.

Considering cost-efficacy, MCT is a prominent therapeutic option (Bocci et al., 2005). Usually, generic, non-patented drugs are used in MCT applications. Many of these drugs can also be supplied in oral forms, which are easier to administer and require no hospital care during the administration process (André et al., 2013). The toxicity profile of MCT is profoundly less than that of other regimens, especially MTD (Benzekry and Hahnfeldt, 2013). This eliminates the need for costly hospital stays for the inevitable normal tissue recovery phase after the treatment (C). The minimal toxicity-inducing doses of MCT induce fewer adverse effects and decrease the cost of monitoring as well as supporting care (Munzone and Colleoni, 2015). All of the above-mentioned reasons make MCT a feasible potential therapeutic option in the fight against cancer.

A tremendous amount of data has been produced in cancer research, and the accumulation of data has continued over the years. One beneficial way to make use of this data is via the utilization of mathematical models of cancer growth and treatment (Araujo and McElwain, 2004; Tabassum et al., 2019). Using mechanistic insights and better understanding of cellular parameters provided by those mathematical models, both clinicians and biologists have a chance to develop better strategies to combat cancer (Majumder and Mukherjee, 2007; Gallasch et al., 2013). Mathematical modeling in cancer research dates back to the 20th century with the majority of these studies focusing on the tumor growth itself (Moolgavkar, 1991). The temporal administration of drugs has been an important area of interest within those efforts. For example, West and Newton designed an evolutionary growth/regression model to investigate the effects of chemotherapeutic dose scheduling (West and Newton, 2017). They compared the efficacy of MCT and MTD chemotherapeutic regimens on various tumors bearing different growth rates. They concluded that MCT strategies exceeded MTD in terms of a decrease in the total number of tumor cells, especially in the fast-growing tumors. Another study performed by Curtis et al., proposed a pharmacokinetic/pharmacodynamic model coupled with a vascularized tumor growth model to stimulate MTD and MCT regimens in lung cancer chemotherapy cases. They concluded that metronomic regimens were more advantageous than MTD regimens, and the combination of these two strategies did not improve the outcome (Curtis et al., 2018). Due to the heterogeneous nature of tumor populations, mathematical models of various kinds have been proven to favor low-dose employing regimens, such as MCT over MTD in this context (Ledzewicz and Schättler, 2014).

## 2 Metronomic chemotherapy: mechanisms of action

**FIGURE 2:**
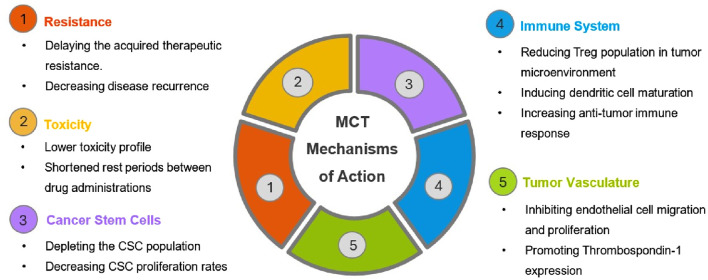
Schematic representation of mechanisms of action of metronomic chemotherapy.

When MCT was first discovered, it was thought that the main function of this treatment was anti-angiogenesis (Colleoni et al., 2002; Perroud et al., 2013). As more evidence accumulated through both preclinical studies and clinical data, it was later revealed that MCT had not only provided anti-angiogenetic properties but also various other functions (Cazzaniga et al., 2016; Romiti et al., 2017). The aspects of metronomic chemotherapy highlighted in the review herein are summarized in Figure 2. In this section, important advantages and the main mechanisms of action of MCT will be discussed in detail, such as decreasing angiogenesis and increasing anti-tumor immune response.

### 2.1 Metronomic therapy induces less acquired therapeutic resistance

Intertumoral and intratumoral heterogeneity have been a hot topic of research in the past years (Liu et al., 2018; Bergmann et al., 2020; Yalcin et al., 2020; Grzywa et al., 2021). The tumor heterogeneity contributing to the presence of one or more cancer cell clones that are resistant to the chemotherapeutic agent of choice could be present prior to the treatment or it might emerge during the course of the treatment (Kelderman et al., 2014; Danisik, et al., 2023; Yalcin et al., 2023; Baygin et al., 2024). The heterogeneous nature of the tumor population contributes to the latter; acquired therapeutic resistance and poses a serious threat to the therapeutic outcomes (Foo and Michor, 2014; Acar et al., 2020). One of the key differences between MCT and MTD treatment regimens is the extent of their ability to form acquired therapeutic resistance since the temporal administration status of drugs is a key determinant of their resistance profile (Goldman et al., 2015; Kareva et al., 2015). As previously discussed, MTD administration generally results in increased acquired therapeutic resistance. The MTD therapy aims to eliminate all drug-sensitive cells through employing the highest possible dose of the cytotoxic agent, this in return creates a selective pressure leading to the selection of the most fit’’ drug-resistant clones in the tumor population (Foo and Michor, 2010; Vasan et al., 2019). Even though most of the drug-sensitive clones have been eliminated, this phenomenon drives the rapid recolonization of the tumor population through drug-resistant clones, hence relapses arise (Dagogo-Jack and Shaw, 2018). In the case of MCT, the observed effect of acquired therapeutic resistance is significantly lower than that of MTD (Scharovsky et al., 2020; Patwardhan et al., 2021). Typical MCT regimens employ much lower doses than MTD, and in return ensure the maintenance of the drug-resistant tumor population since the selective pressure is not as prominent (Bondarenko et al., 2021). A recent study performed by Mejia Peña et al. (2023), demonstrated that the administration of metronomic or MTD Paclitaxel regimens resulted in different resistant cancer cell populations with unique nuclear and phenotypic traits. The evidence they provided pointed toward the phenomenon that these two different resistant populations are primed differentially for the response they might give towards chemotherapy or induced metastasis stress (Meja Peña et al., 2023). In conclusion, metronomic chemotherapy is emerging as a prominent treatment strategy, offering the potential to mitigate acquired therapeutic resistance.

### 2.2 The low toxicity profile of metronomic chemotherapy

The toxicity induced by chemotherapy is considered as one of the most important side effects (Livshits et al., 2014). The working mechanism of the chemotherapeutic drugs is to damage proliferating cells through inducing DNA damage, impairing mitosis and DNA damage repair mechanisms, and eventually leading to apoptosis (Kaufmann and Earnshaw, 2000; Hayashi and Karlseder, 2013). Importantly, these damages are not specific to the cancer cells but rather they affect all proliferating cells, including the normal ones (Toale et al., 2021). The cytotoxic burden of the treatment affects the administration intervals to allow sufficient time for the recovery of damaged non-cancerous tissues. MTD requires prolonged rest periods between the administrations since it utilizes highly cytotoxic doses. On the contrary, the MCT regimen has been shown to exhibit a much lower toxicity profile than MTD (Emmenegger et al., 2004; Simsek et al., 2019; Cazzaniga et al., 2022). The lower cytotoxic burden also means fewer side effects and an increased quality of life for the patients in general (Xu et al., 2021; Wysocki et al., 2022). This is especially advantageous for cancer patients in palliative care as the expectations of the treatment outcome are symptomatic relief and low toxicity (Patil et al., 2019; Harsh et al., 2023). In summary, the low toxicity profile of metronomic chemotherapy renders it an appealing and viable treatment option for cancer of many kinds.

### 2.3 Effects of metronomic chemotherapy on the immune system

The immune system plays a double-edged role in the initiation, progression and metastasis stages of cancers (Visser et al., 2006; NarendraBodduluru et al., 2013). Hanahan and Weinberg have categorized tumor-promoting inflammation and avoiding immune destruction as two of the hallmarks of cancer in their seminal 2011 paper (Hanahan and Weinberg, 2011). Among the many cells of the immune system, regulatory T cells (T-regs) are of great importance in the context of the tumor microenvironment (Wolf et al., 2015; Paluskievicz et al., 2019). T-regs are a specialized subset of helper T lymphocytes that bear the important role of modulating the immune response (Shevyrev and Tereshchenko, 2020). They express specific cell surface markers such as CD3, CD4, and CD25, as well as FoxP3 (Tanaka and Sakaguchi, 2019). They are important for building tolerance towards the host’s self-antigens, and they take part in key cellular processes including allergies, tumorigenesis, neoplasia and infections (Martins et al., 2012). Dysfunction of T-reg cells leads to an over-suppression of the immune response and it is correlated with a number of different diseases such as autoimmune disorders, allergy and cancer (Attias et al., 2019; Ohue and Nishikawa, 2019). Within the context of cancer, T-reg cell overexpression poses a serious problem as it results in the over-suppression of the effector anti-tumor immunity which may result in treatment unresponsiveness and tumor progression. Also, the assembling of T-reg cells in tumor sites as well as in the peripheral circulation leads to immune evasion of the tumors (Frydrychowicz et al., 2017). Furthermore, the anti-tumor immune response significantly weakens as the T-reg cells secrete cytokines (IL-10, TGF-β etc.) that suppress other types of immune cells such as helper and cytotoxic T cells, macrophages, natural killer cells that are responsible for anti-tumor responses (Ohue and Nishikawa, 2019). Although, it should be noted that the presence of T-reg cells is of great importance for numerous other vital functions, such as fighting autoimmunity, hence, the required T-reg population should be maintained (Wolf et al., 2015).

The administration of various chemotherapeutic agents has been reported to induce T-reg-decreasing effects. Banissi et al. has reported that a metronomic regimen of Temozolomide was the most successful among other treatment regimens in decreasing the T-reg population in a rat glioma model (Banissi et al., 2009). Another study performed by Shevchenko et al. investigated the effects of metronomic Gemcitabine administration on murine pancreatic cancer model Panc02. They concluded that the administration of the metronomic Gemcitabine depleted the T-regs, and it resulted in improved survival of the animal models (Shevchenko et al., 2013). In particular, the Treg-related effects of the metronomic administration of the drug Cyclophosphamide has been extensively studied in both animal models and clinical trials. Ge et al. reported that metronomic administration of Cyclophosphamide led to a selective and rapid decrease in the T-reg cell numbers of breast cancer patients and noted that this decrease was temporary (Ge et al., 2012). Generali et al. reported that Cyclophosphamide in combination with the aromatase inhibitor Letrozole resulted in a significant decrease in T-reg population in a cohort of 83 breast cancer patients (Generali et al., 2009) Ghiringhelli et al. has also shown that metronomic oral administration of Cyclophosphamide has led to a selective decrease in circulating T-reg cells, which positively correlated with the therapeutic outcomes of advanced cancer patients (Ghiringhelli et al., 2007).

Apart from decreasing the immune suppression caused by T-reg cells, MCT has been shown to have promising effects on enhancing the anti-tumor immune response as well. Cytotoxic T cells are a subset of immune cells that represent one of the most potent effectors in the immune response against cancer. They can be distinguished by their expression of the CD8 cell surface marker and serve as primary destroyers of pathogens as well as tumor cells (Raskov et al., 2021). Increase in the cytotoxic T-cell population was also observed in many MCT studies. Hermans et al. asserted that metronomic administration of Cyclophosphamide resulted in a considerably larger increase in the cytotoxic T-cell population compared to the MTD administration in a mouse model. They also observed that the MTD administration of Cyclophosphamide caused a dramatic decrease in the overall cytotoxic T-cell population after 28 days, which was not the case in the MCT administration of the same drug (Hermans et al., 2003). Likewise, He et al. reported that metronomic administration of Paclitaxel in combination with an anti-cancer vaccine has significantly increased the cytotoxic T-cell infiltration into the tumor tissue in a prostate cancer mouse model. In the same study, they also observed increased dendritic cell maturation, translating into enhanced anti-tumor immune response (He et al., 2011). Dendritic cells are another subset of immune cells that are responsible for activating the T-cell-mediated anti-tumor response through their antigen presenting properties (Wculek et al., 2020). Finally, additional studies have also supported the anti-tumor immune response-promoting effects of MCT (Ghiringhelli et al., 2007; Ge et al., 2012; Khan et al., 2020). Altogether, the presented data strongly indicate that metronomic chemotherapy not only diminishes the suppressive impact of regulatory T cell populations but also enhances the anti-tumor immune response, underscoring its potential as a pivotal element in cancer treatment strategies.

### 2.4 The anti-angiogenesis effects of metronomic chemotherapy

The cells in the human body require certain nutrients and oxygen to survive. To supply this demand, they must be located near blood vessels, the highways of the body, and new blood vessels should be recruited through processes called vasculogenesis and angiogenesis as the organism gets larger (Nishida et al., 2006). Angiogenesis refers to the formation of new capillaries through the pre-existing blood vessels. It is part of normal cellular processes such as wound healing and embryogenesis as well as pathological conditions such as atherosclerosis, tumorigenesis and rheumatoid arthritis (Rajabi and Mousa, 2017). In the case of tumors, the formation of new blood vessels is especially important for the tumor to grow to a certain size and for the metastasis process, moreover, it has been defined as one of the hallmarks of cancer (Hanahan and Weinberg, 2011). Pro-angiogenic and anti-angiogenic molecules are responsible for regulating this crucial cellular process. These molecules can be influenced by various events such as hypoglycemia, inflammatory response, and genetic changes, and they can originate from endothelial cells, blood, and cancer cells (Huang and Bao, 2004). Among the pro-angiogenic molecules, vascular endothelial growth factor (VEGF), fibroblast growth factor-2 (FGF-2), platelet-derived growth factor (PDGF), and angiopoietins have been characterized. On the other hand, thrombospondins TSP-1 and TSP-2, angiostatin, interleukins IL-1 β, IL-3, IL-6, IL-8, and tumor necrosis factor (TNF) α are crucial effector molecules of anti-angiogenesis (Carmeliet and Jain, 2000). Altogether, the balance between these two classes of molecules is of utmost significance for angiogenesis capacity and tumor control. If this balance is disrupted, favoring the pro-angiogenic side, the “angiogenic switch’’ occurs (Bergers and Benjamin, 2003). This results in circulating endothelial progenitors (CEP) recruitment which are able to promote angiogenesis. In addition, the angiogenic switch can induce genetic reprogramming in the cancer cells, consequently inducing new blood vessel formation, all of which favor tumor progression (Natale and Bocci, 2018).

The earliest studies on metronomic therapy have showcased the anti-angiogenesis effects of the treatment option (Browder et al., 2000; Klement et al., 2000). Since then, it has been assumed that the main anti-tumor effect of MCT was its anti-angiogenesis properties. This is postulated to be due to the characteristics of the tumor endothelial cells and the mechanism of action of the chemotherapeutic agents (Kerbel and Kamen, 2004). In the MCT regimens, low doses of the chemotherapeutic drugs are employed in a continuous manner. This ensures the presence of a certain level of the drug in the body for longer times. Chemotherapeutic drugs are not selective by nature; they act by disrupting the cellular proliferation of all dividing cells, including the endothelial cells found in growing blood vessels around the tumor sites (Soultati et al., 2012). Endothelial cells are relatively more genetically stable than cancer cells and do not develop drug resistance as easily (Hida et al., 2018). Therefore, the presence of chemotherapeutic drugs without long rest periods ensures the elimination of the dividing endothelial cells around the tumor, thus, translating into anti-angiogenic effects (Kerbel and Kamen, 2004). On the other hand, longer rest periods are needed in MTD regimens, thus, giving endothelial cells around the tumor more time to recover from the detrimental chemotherapeutic effects.

One of the pioneering studies on the anti-angiogenic effects of metronomic chemotherapy was performed in 2003 by Wang et al. They have demonstrated that when the drug Paclitaxel was administered at low doses (0.1–100 p.m.), it selectively inhibited the human endothelial cell proliferation *in vitro*. Whereas, when they administrated the same drug at concentrations 10^4–^10^5^ folds higher, they observed the inhibition of cells that are of non-endothelial origin (Wang et al., 2003). Perroud et al. has analyzed the serum levels of angiogenesis markers in breast cancer patients undergoing MCT treatment of Cyclophosphamide and Celecoxib. They have demonstrated that the ratio of VEGF/TSP-1 has decreased significantly during the course of the therapy. This suggests that there has been a shift towards the anti-angiogenic molecule TSP-1 during the treatment (Perroud et al., 2013). Similarly, Colleoni et al. has also looked at the serum VEGF levels of 63 breast cancer patients that received MCT treatment of Methotrexate and Cyclophosphamide. They displayed a decrease in VEGF levels, therefore, a decrease in angiogenesis after the MCT treatment (Colleoni et al., 2002). A recent study performed by Mundo et al., investigated the differences between angiogenesis markers on a mouse colorectal cancer model treated with Fluorouracil following either a MTD or MCT regimen. They have shown that the MTD regimen leads to a more prominent increase in the mean tissue oxygen saturation and oxyhemoglobin levels. In addition, the MTD group had a sustained decrease in VEGF expression. It was concluded that the MTD group displayed more evident blood vessel remodeling, which is correlated with angiogenesis (Mundo et al., 2022). To sum up, altering angiogenesis was the first effective mechanism of MCT to be identified, and until this day, a growing body of data confirm that MCT is responsible for decreasing angiogenesis (Pietras and Douglas, 2005; Biziota et al., 2017; Natale and Bocci, 2018).

### 2.5 Cancer stem cell (CSC) targeting effects of metronomic chemotherapy

Cancer stem cells (CSCs) are a subpopulation of cells harboring cancer-initiating properties (Wysocki et al., 2022). The CSCs were first identified in 1994, when a subgroup of cancer cells was isolated through their CD34^+^ and CD38^−^ surface marker expressions, in a study performed on a leukemia mouse model (Lapidot et al., 1994). They are also distinguished by expression signature of the transcription factors OCT4, Sox2, MYC, Nanog, and signaling pathways Wnt, Notch, JAK-STAT (Yang et al., 2020). Other biomarkers of CSC include expression of surface markers CD44, CD133, CD166 and CD90 (Leon et al., 2016). The CSCs are characterized by their self-renewal and cell differentiation capacities, thereby playing a fundamental role in various aspects of tumor malignancy. This subpopulation of cancer cells is especially important in the biological processes of tumor recurrence and metastasis, owing to their differentiation, senescence and self-renewing properties. Accordingly, CSCs pose a serious threat to the efficacy of cancer therapies. Several studies have shown that MCT led to a decrease in the proliferation and survival rates of CSCs (Folkins et al., 2007; Muñoz et al., 2021; Wysocki et al., 2022). In an *in vitro* study, MCT administration of Paclitaxel has been shown to reduce CSC population (Salem et al., 2020). In another study, the CSC population-depleting effects of MCT administration of Cyclophosphamide was shown in a xenograft model of pancreatic cancer (Vives et al., 2013). To conclude, beneficial effects of MCT on decreasing the CSC population, an important player in cancer progression, have been demonstrated by several studies.

## 3 The translation of MCT: from Petri dish to the clinic

The effects of various MCT regimens as first- and second-line treatments have been extensively studied in the clinic. Numerous ongoing phase II and III clinical trials persist in elucidating the diverse effects of metronomic chemotherapy on patients in real-world scenarios. Among these, the clinical studies concerning breast cancer patients are especially high (Montagna et al., 2017; Lago et al., 2022; Chai et al., 2023). In 2017, The International Consensus Guidelines for Advanced Breast Cancer (ABC) affirmed that MCT is a reasonable treatment for patients without the need for rapid tumor shrinkage. They also stated that so far, especially the low-dose oral Cyclophosphamide and Methotrexate MCT regimen had accumulated substantial clinical data and called for more randomized trials to better compare MCT with other standard regimens. The published statement garnered agreement from a consensus of 88% of the panelists (Cardoso et al., 2017). MCT is also a popular treatment option among clinical studies regarding other types of cancer, such as prostate cancer, ovarian cancer and non-small cell lung cancer (NSCLC). Table 1 provides a detailed overview of selected clinical studies conducted over the past decade, encompassing phase II, phase III, and retrospective studies across various cancer types. Overall survival (OS), progression free survival (PFS) and objective response rate (ORR%) properties were displayed to assess the efficacy of the therapy choice.

**TABLE 1:** Selected clinical studies concerning metronomic chemotherapy.

Study design	Cancer type	Metronomic regimen	Patients (n)	Median PFS	Median OS	ORR %	References
phase I/II	oral Cancer	metronomic erlotinib, methotrexate, and celecoxib	91	71.10%	61.20%		Patil et al. (2019)
phase II	breast cancer	metronomic vinorelbine, cyclophosphamide and capecitabine	25	34% after 1 year		27%	Montagna et al. (2017)
phase II	breast cancer	metronomic oral vinorelbine and capecitabine	29	12.5 months		31.00%	Chai et al. (2023)
phase II	breast cancer	metronomic capecitabine and cyclophosphamide	51	12.3 months	86%, after 1 year	44.40%	Yoshimoto et al. (2012)
phase II	breast cancer	Metronomic capecitabine combined with aromatase inhibitors	44	16.2 months		70.5%	Li et al. (2019)
phase II	ovarian cancer	pembrolizumab, bevacizumab, and oral cyclophosphamide	40	10 months		47.50%	Zsiros et al. (2021)
phase II	breast cancer	Metronomic trastuzumab, oral capecitabine and cyclophosphamide	66	47.7% after 1 year	45.9 months	56.70%	Orlando et al. (2020)
phase II	NSCLC	oral vinorelbine	43		9 months	18.60%	Camerini et al. (2015)
phase II	breast cancer	Arm A: vinorelbine Arm B: vinorelbine, capecitabine	120	7.1 months in arm A and 6.3 months in arm B	23.3 months in arm A and 22.3 months in arm B	24% in arm A and 29% in arm B	Brems-Eskildsen et al. (2020)
phase II	ovarian cancer	Arm A: oral metronomic etoposide, cyclophosphamide Arm B: Pazopanib	75	3.4 months in arm A and 5.1 months in arm B	11.2 months in arm A, not reached In arm B		Sharma et al. (2021)
phase II	pediatric solid tumours	Nivolumab, oral metronomic cyclophosphamide	13	1.7 months	3.4 months		Pasqualini et al. (2021)
phase II	NSCLC	cisplatin, metronomic oral vinorelbine	65	11.5 months	35.6 months	78.48%	Provencio et al. (2021)
phase II	breast cancer	oral metronomic vinorelbine	9	12.0 weeks		38%	Krajnak et al. (2021)
phase II	breast cancer	fulvestrant, oral capecitabine	41	14.98 months	28.65 months	24.40%	Schwartzberg et al. (2014)
phase II	breast cancer	metronomic cyclophosphamide, capecitabine, vinorelbine	108	6.9 months	91% in naive patients and 83% in pre-treated patients		Montagna et al. (2017)
phase II	Medulloblastoma	oral metronomic thalidomide, fenofibrate, celecoxib, alternating cycles of oral etoposide and cyclophosphamide	40	8.5 months	25.5 months		Peyrl et al. (2023)
phase II	ovarian cancer	metronomic cyclophosphamide and temozolomide	55	5.9 months	10.1 months	44%	Bhattacharyya et al. (2015)
phase II	breast cancer	trastuzumab plus pertuzumab or trastuzumab and pertuzumab plus metronomic oral cyclophosphamide	80	46.2% vs. 73.4%			Lago et al. (2022)
phase II	ovarian, fallopian tube or primary peritoneal cancer	metronomic cyclophosphamide and nintedanib	117	2.9 months	6.8 months		Hall et al. (2020)
phase II	breast cancer	metronomic oral vinorelbine and trastuzumab	20	7.4 months	45.8 months	20.00%	Wang et al. (2021)
phase II	NSCLC	cisplatin, metronomic oral vinorelbine	65	11.5 months	35.6 months	78.48%	Provencio et al. (2021)
phase II	breast cancer	oral metronomic vinorelbine	9	12.0 weeks		38%	Krajnak et al. (2021)
phase II	breast cancer	fulvestrant, oral capecitabine	41	14.98 months	28.65 months	24.40%	Schwartzberg et al. (2014)
phase II	breast cancer	metronomic cyclophosphamide, capecitabine, vinorelbine	108	6.9 months	91% in naive patients and 83% in pre-treated patients		Montagna et al. (2017)
phase II	Medulloblastoma	oral metronomic thalidomide, fenofibrate, celecoxib, alternating cycles of oral etoposide and cyclophosphamide	40	8.5 months	25.5 months		Peyrl et al. (2023)
phase II	ovarian cancer	metronomic cyclophosphamide and temozolomide	55	5.9 months	10.1 months	44%	Bhattacharyya et al. (2015)
phase II	breast cancer	trastuzumab plus pertuzumab or trastuzumab and pertuzumab plus metronomic oral cyclophosphamide	80	46.2% vs. 73.4%			Lago et al. (2022)
phase II	ovarian, fallopian tube or primary peritoneal cancer	metronomic cyclophosphamide and nintedanib	117	2.9 months	6.8 months		Hall et al. (2020)
phase II	breast cancer	metronomic oral vinorelbine and trastuzumab	20	7.4 months	45.8 months	20.00%	Wang et al. (2021)
phase II/III	esophageal squamous cell carcinoma	metronomic celecoxib and methotrexate	151	25 months	36 months		Noronha et al. (2022)
phase III	breast cancer	metronomic methotrexate and cyclophosphamide	158	26 months	33 months		Nasr et al. (2015)
phase III	nasopharyngeal carcinoma	metronomic oral capecitabine	406	85·3% (FFS)			Chen et al. (2021)
phase III	head and neck carcinoma	oral netronomic methotrexate and celecoxib	422	3·23 months	7.5 months		Patil et al. (2020)
phase III	pediatric cancers	oral celecoxib, thalidomide, and fenofibrate, with metronomic cyclophosphamide and etoposide	97		60%		Robison et al. (2014)
retrospective	NSCLC	osimertinib with metronomic oral vinorelbine tartrate	28	9.4 months		17.90%	Li et al. (2023)
retrospective	breast cancer	metronomic oral cyclophosphamide and methotrexate	120	12.0 weeks			Krajnak et al. (2020)
retrospective	breast cancer	fulvestrant with metronomic polychemotherapy VEC (vinorelbine, cyclophosphamide and capecitabine)	39	8.4 months	21.5 months		Buda-Nowak et al. (2023)
retrospective	breast cancer	metronomic vinorelbine	90			65.50%	Liu et al. (2021)
retrospective	breast cancer	metronomic oral cyclophosphamide and methotrexate, CTX and capecitabine, CTX, or vinorelbine alone	72	17.0 weeks	58.0 weeks		Krajnak et al. (2023)
retrospective	breast cancer	metronomic cyclophosphamide, capecitabine, etoposide and vinorelbine, alone or in combinaton	584	6.28 months	22.7 months in VRL-only, 30–14.2 months in other regimens		Cazzaniga et al. (2019)
retrospective	ovarian cancer	metronomic oral etoposide, cyclophosphamide and tamoxifen	40	3.7 months	6.5 months	60%	Harsh et al. (2020)
retrospective	prostate cancer	cyclophosphamide and prednisolone	14		8.1 months		Knipper et al. (2019)
retrospective	hepatocellular cancer	capecitabine	117		8 months		Granito et al. (2015)
retrospective	prostate cancer	oral metronomic cyclophosphamide	74	4.0 months	8.1 months		Caffo et al. (2019)
retrospective	prostate cancer	metronomic cyclophosphamide and low dose of corticosteroids	37	11 months	28 months		Calvani et al. (2019)
retrospective	prostate cancer	metronomic oral cyclophosphamide with or without oral prednisolone	18	4.7 months			Dabkara et al. (2018)
retrospective	prostate cancer	metronomic cyclophosphamide, etoposide, estramustine, ketoconazole and prednisolone	123	4.4 months	12.3 months		Asowed et al. (2023)
retrospective	ovarian cancer	topotecan or topotecan and metronomic cyclophosphamide	72	3.65 months		27%	Wysocki et al. (2023)
retrospective	ovarian cancer	metronomic cyclophosphamide, etoposide and celecoxib with or without pazopanib	36	8.2 months	38 months	19%	Sharma et al. (2019)

## 4 Combination therapy strategies regarding MCT

Combination therapeutic approaches have been widely used in cancer therapy. When compared with mono-therapy, the efficacy of the treatment is often increased synergistically or in an additive fashion when two or more therapeutic agents are used in combination (Matlock et al., 2017; Mokhtari et al., 2017). This phenomenon occurs primarily because approaches targeting distinct pathways are employed together in combination therapy. Consequently, the risk of developing acquired resistance to treatment and the subsequent progression of the tumor is reduced (Leary et al., 2018).

For a study design of anti-cancer combination therapy, the toxicity aspect is of great concern, since when more than one cytotoxic agent is being utilized it may result in an increased toxic burden on the patient (Delbado et al., 2004; Parhi et al., 2012). MCT is commonly used in combinational therapeutic approaches, primarily owing to its low toxicity profile and its capacity to minimize the acquired therapeutic resistance. There are many studies in which MCT was employed in combination with other anti-cancer therapies such as immunotherapy (Soriano et al., 2011), radiation therapy (Chung et al., 2017) and oncolytic virotherapy (Liikanen et al., 2013). There are also cases where MCT is coupled with an MTD treatment approach of another chemotherapeutic agent to strengthen the anti-tumor response without inducing adverse toxicity (Brems-Eskildsen et al., 2021).

Radiation therapy is a commonly used treatment modality for different cancer types. Approximately 50% of all cancer patients undergo radiation therapy as a part of their treatment regimen throughout the course of their illness (Baskar et al., 2012). A number of studies reported promising results upon the administration of combination therapy regimens employing radiation therapy along with MCT (Štěrba et al., 2002; Choi et al., 2008; Chung et al., 2017). It has been shown that the metronomic administration of four different drugs; namely, Celecoxib, Vinblastine, Cyclophosphamide, and Methotrexate coupled with radiotherapy afterward, resulted in favorable outcomes in 64 pediatric solid tumor-bearing patients (Ali and Mohamed, 2016). The disease response rate was found to be ∼76.6%, coupled with minimal toxicity and a 62% 1-year overall survival rate (Ali and Mohamed, 2016).). In another study, Salmaggi et al. investigated the effects of a combination of Carmustine wafers (which are implanted into the brain to release the drug Carmustine as the wafers dissolve), metronomic Temozolomide and radiotherapy regimen for the treatment of glioblastoma patients. They compared the efficacy of the treatment as compared to the Stupp protocol, a standard practice for the treatment of newly diagnosed glioblastoma patients (Salmaggi et al., 2013). They reported an increase in progression-free survival without the increased toxicity, as compared with the standard Stupp regimen. However, they also noted that the median survival rate increase in their newly-formed schedule was not as evident as in Stupp schedule, and therefore prospective comparative trials were recommended (Salmaggi et al., 2013). Collectively, combination therapeutic strategies employing radiation therapy along with metronomic chemotherapy have been shown to be effective in many studies.

Oncolytic virotherapy is a form of cancer therapy utilizing oncolytic viruses that specifically replicate and damage cancerous cells (Davis and Fang, 2005). It offers a multidirectional therapeutic platform since the viral vectors can be engineered to harbor transgenes that alter their cytotoxicity and immunostimulatory properties (Harrington et al., 2019). Combination therapies consisting of oncolytic virotherapy along with metronomic administration of chemotherapeutic drugs have been reported to have increased efficacy (Bramante et al., 2016; Toulmonde et al., 2022). Qiao et al. reported that the metronomic administration of a combination of Cyclophosphamide along with oncolytic reovirus resulted in greater antitumor efficacy and less toxicity when compared to single high or low-dose administrations (Qiao et al., 2008). Onimaru et al. also showed that the combination therapy involving metronomic administration of Gemcitabine along with engineered adenovirus resulted in increased cytotoxicity of cancer cells in pancreatic cancer mouse models. They have attributed this increase to the crosstalk mechanisms between the two components, allowing for enhanced viral entry and subsequent cancer cell cytotoxicity (Onimaru et al., 2010). A similar case of enhanced oncolytic adenovirus anti-tumor efficacy was reported when combined with low-dose Paclitaxel (Ingemarsdotter et al., 2015). Another study performed by Cheema et al. has also demonstrated that metronomic etopside induction enhanced the antitumor properties of oncolytic herpes simplex virus treatment and decreased the cytotoxic burden of the viral treatment on human glioblastoma stem cell xenografts (Cheema et al., 2011). Taken together, these data suggest that the combination of virotherapy with MCT is a promising strategy to achieve increased anti-tumor properties as well as decreased toxicity.

Cyclophosphamide is a widely used chemotherapeutic drug for the treatment of neoplasms, it is a nitrogen mustard and it induces anti-tumor effects through alkylation (Emadi et al., 2009; Voelcker et al., 2020). While relatively successful at eradicating the tumor cells, Cyclophosphamide is known to have a high cytotoxic profile, limiting its utilization (Haubitz, 2007). Rather than the highly cytotoxic MTD administration, MCT administration of Cyclophosphamide is extensively being utilized in many studies in combination with various other therapies such as vaccine administrations or immunotherapies (Soriano et al., 2011; Weir et al., 2014). It has been shown to help decrease the immune suppression caused by the tumor and strengthen the anti-immune responses (Hermans et al., 2003; Borch et al., 2016). It has been shown that low-dose metronomic application of Cyclophosphamide strengthens both the adaptive and innate anti-tumor immune responses when combined with a TLR9 agonist (Leong et al., 2019). In another study, Webb et al. proposed that a combination of anti-PD1 immunotherapy and metronomic CPA administration resulted in an increased survival rate among mouse models (Webb et al., 2022).

Lastly, drug repurposing is another phenomenon worth mentioning that helps salve the expenses of novel therapeutic drug research and makes MCT an attractive option. Drug repurposing is a remarkably cost-effective approach to utilize old drugs for novel purposes (Sleire et al., 2017; Correia et al., 2021). It enables faster clinical translation since the time-consuming testing of parameters such as toxicity, efficacy, and pharmacodynamic properties of the drug are already set. MCT offers a new possibility for the repurposing of old, non-cancer drugs. Muscarinic agonists are a great example of popular drug repurposing efforts in the context of MCT (Sales, 2018; Salem et al., 2020). In addition, since MTD therapy has been the cornerstone of chemotherapeutic treatment for a long time, most of the chemotherapeutic drugs were tested specifically on MTD regimen. MCT offers a promising novel low-cost option for the use of already existing chemotherapeutic drugs, as it has been proven by many studies that MCT and MTD regimens of the same drug induce different biological effects (Tongu et al., 2013; Fares et al., 2020).

## 5 More than chemotherapy: Metronomic photodynamic therapy

Photodynamic therapy (PDT) is a new field of cancer therapy. It is based on the principle of utilizing photosensitizer drugs which are administrated to the tumor area, and then irradiated when light is induced into the area (Agostinis et al., 2011). As the photosensitizer drug absorbs either visible or near-infrared light, a series of photochemical reactions are then triggered, releasing cytotoxic reactive oxygen species (ROS) byproducts and leading to cancer cell death through free radical-related DNA damage, oxidative stress, apoptosis, or necrosis (Wilson and Patterson, 2008; Kwiatkowski et al., 2018). PDT treatment has been a popular choice for the treatment of skin cancers owing to the feasibility of the treatment related to the positioning of the tumor, as well as brain cancers since modern therapies have been very limited (Quirk et al., 2015).

A downside of PDT was the emergence of treatment resistance due to the rapid consumption of oxygen that comes with using a high fluence rate. The concept of metronomic photodynamic therapy (mPDT) emerged around 2 decades ago, meeting the need for decreased treatment resistance and lower toxicity profile (Bisland et al., 2004). Metronomic photodynamic therapy is based on both the photosensitizer drug and the light source being metronomically applied at a low rate, continuously to maximize specifically tumor cell ablation and minimize damage to normal tissues. Especially in the case of brain cancer treatment, choosing a regimen inducing low toxicity to normal brain tissues is of utmost importance. In one of the earliest works within this research area, Davies and Wilson examined the feasibility of metronomic application of the photosensitizer 5-aminolevulinic acid induced protoporphyrin IX (ALA-PpIX) mediated photodynamic therapy in a rat-derived astrocytoma model. They were able to demonstrate a 4-day metronomic delivery, and reported that mPDT was the most successful delivery option among other PDT regimens. They also observed the ablation of the newly formed pre-bulk tumor population through metronomic application (Davies and Wilson, 2007). Other than brain tumors, the potential of mPDT has been demonstrated in various other types of cancers, such as colorectal cancer and cervical cancer (Shi et al., 2019; Dai et al., 2023) In a study by Caverzán et al., standard and metronomic regimens of conjugated polymer nanoparticles-based PDT was assessed on glioblastoma cell lines and xenograft mouse models. They reported that mPDT regimes induced increased cancer cell death, and lowered therapeutic resistance furthermore resulted in the polarization of one of the most important cancer-related immune cell subtypes, macrophages, towards an anti-tumoral phenotype (Caverzán et al., 2023).

There exists an additional challenge that needs to be overcome in the application of mPDT; delivery systems enabling the metronomic induction of the treatment. A growing interest has been towards the construction of wireless devices that adhere to the tissue of interest and ensure the induction of PDT in a metronomic manner (Lin et al., 2023). Another strategy is utilizing engineered microneedle devices for the delivery of photosensitizers in mPDT (Dai et al., 2023). The nature of this treatment option calls for interdisciplinary cooperation to help it advance. There is a need for optimization of the delivery systems used in metronomic photodynamic therapy.

In addition, the success of metronomic administration of photodynamic therapy underlines one critical point; that metronomic regimens do not have to be limited to chemotherapeutic uses only. The term ‘‘metronomic’’ refers to the type of temporal administration of the therapeutic agent used. As discussed above, the success of metronomic chemotherapy is mainly attributed to its low toxicity profile and its ability to decrease acquired therapeutic resistance. However, toxicity and resistance concerns are not limited to the use of chemotherapeutics, they are key factors to consider within the applications of other treatment options, too. Given that studies concerning metronomic chemotherapy and metronomic photodynamic therapy have both shown similar results in terms of their efficacy, metronomic therapy can be considered a promising temporal regimen that is not only limited to the chemotherapeutic administrations. This promising hypothesis calls for more studies concerning the use of metronomic administration in other therapeutic administrations also.

## 6 Limitations and challenges associated with MCT

As in all therapeutic efforts, MCT presents both merits and drawbacks. Recognizing cancer’s multifaceted and complex nature, the suitability of MCT may not be universal across all cases. Particularly in instances where the tumor burden is high, the MCT approach is usually not prioritized as it does not result in rapid shrinkage of the tumor. MCT may not be very suitable for aggressive tumor types also due to the aforementioned rationale. MCT is considered to be a minimally toxic and less resistance-inducing treatment approach, but it comes with the trade-off of the rapid and potent tumor shrinkage that MTD therapy is typically associated with. It is important to recognize the necessity of implementing precision medicine notion here by tailoring treatment strategies to the specific needs and characteristics of the individual medical cases, appreciating the nuances governing therapeutic decision-making by medical professionals.

Both the terms ‘‘metronomic therapy’’ and ‘‘metronomic chemotherapy’’ are used broadly, and there are no clear boundaries for their descriptions. In light of the success of metronomic PDT, we believe that metronomic therapy should be clearly defined as an umbrella term that refers to a certain administration regimen of cancer therapies of many kinds. Hence, metronomic therapy includes MCT, but it is not limited to chemotherapeutic administration alone. We believe there needs to be a clear distinction between the two terms in the literature.

Furthermore, administration of a chemotherapeutic drug at doses lower than MTD within short intervals is considered MCT, but different outcomes emerge when the drug is given under different parameters. The intervals between drug administrations, the dosage, are all among critical factors contributing greatly to the outcome of the treatment. In addition, as in many other therapeutic cases, when the drug is used in combination with another drug or therapy, the effects differ vastly. In a study performed in 2013, Tongu et al. reported that when Cetuximab was administered at 4-day intervals, it weakened the anti-tumor T cell response in mice models, whereas when the drug was administered at 8-day intervals along with Gemcitabine, an increased sensitivity of T-reg cells was observed (Tongu et al., 2013). This phenomenon highlights the need for the optimization of metronomic chemotherapeutic schedules and calls for more research to be performed to overcome this challenge.

Mathematical modeling is another powerful tool that aids in the optimization of MCT. Efforts made within this area help determine the parameters of MCT administration that give rise to the best outcomes without the need for trial-error and countless individual research efforts. Kweon et al., established a mathematical model to optimize the oral metronomic Doxorubicin regimen. Their model evaluated the relationships of several cases and determined the dose, frequency, formulation, and administration parameters that are optimal from both tumor suppression (pharmacodynamic) and cardiac toxicity (toxicodynamic) aspects of metronomic Doxorubicin administration (Kweon et al., 2022). We firmly believe that the mathematical modeling efforts to optimize metronomic chemotherapeutic administrations as such are key to the advancement of metronomic chemotherapy research in the upcoming era.

## 7 Conclusion and future perspectives

Considerable progress has been made in the treatment of cancer, each with its own set of successes and setbacks. Given the diverse and complex nature of cancer, it is clear that there is no one-size-fits-all treatment plan, which highlights the importance of precision medicine framework and the ongoing need for better therapeutic approaches. Although MTD chemotherapy has demonstrated efficacy in many conditions, there is increasing recognition that, in many cases, it may not only be ineffective but also have the potential to exacerbate the course of the disease when compared to MCT.

A collective amount of evidence from research over the past 2 decades has strengthened the rationale for MCT as a viable treatment option for a variety of cancer types. MCT’s unique benefit, its comparatively low cytotoxicity, opens the door for combinations with other treatment modalities. The efficacy of combining MCT with radiation therapy or immunotherapy indicates that these combinations should be taken into consideration as potential solutions. Furthermore, acquired therapeutic resistance can be efficiently managed with the metronomic administration of treatment modalities beyond standard chemotherapy, as exemplified by metronomic photodynamic therapy.

It is our opinion and firm belief that metronomic therapy must be viewed as an umbrella term and should not be limited to chemotherapeutic administrations (MCT) alone rather applicable to numerous regimens of cancer therapies of several types. Subsequently, this ought to be reflected in the literature.

In conclusion, MCT has many benefits, including cost-effectiveness, favorable toxicity and resistance profiles, and characteristics that support anti-tumor immune responses and anti-angiogenesis. These discoveries have opened up new therapeutic possibilities, which emphasize the significance of additional biological cancer research, clinical trials, and mathematical modeling approaches. These efforts are essential to thoroughly investigate and leverage on the possible advantages provided by MCT, impacting the course of cancer treatment in the future.
